# Is Cancer Hereditary?

**Published:** 1886-03

**Authors:** 


### Is Cancer Hereditary?

We rather dissent from the view that all diseases can be and no disease need be hereditary; that is to say that a progenitor transmits to his or her offspring more or less of a tendency towards any disease that may exist in him or herself, but that there is a hygiene of every disease, by the observance of which this tendency may be rendered latent, and ultimately eradicated, and vice versa. However, this broad view of heredity is hardly yet universally accepted, and the question of heredity in individual diseases is oftentimes discussed. Dr. Herbert Snow entertained the last meeting of the British Medical Association with a discussion of the heredity of cancer, and thus formulated his views:

1. That the belief in heredity is derived merely from popular tradition, and is wanting in any sound basis or scientific proof.

2. That extremely practical issues are involved, and that the views now prevalent lead to disastrous results—*Med. and Surg. Reporter*.

